# Varicella Infection Complicated by Group A Beta-Hemolytic Streptococcal Retropharyngeal Abscess

**DOI:** 10.1155/2016/9298143

**Published:** 2016-08-29

**Authors:** Christine M. Clark, Colin Huntley, Michele M. Carr

**Affiliations:** ^1^College of Medicine, The Pennsylvania State University, 500 University Drive, Hershey, PA 17033, USA; ^2^Department of Otolaryngology-Head & Neck Surgery, Thomas Jefferson University, 925 Chestnut Street, 6th Floor, Philadelphia, PA 19107, USA; ^3^Division of Otolaryngology-Head and Neck Surgery, Department of Surgery and Department of Pediatrics, College of Medicine, The Pennsylvania State University, 500 University Drive, Hershey, PA 17033, USA

## Abstract

An unimmunized 19-month-old child presented with a retropharyngeal abscess and coincident varicella infection. The abscess resolved with operative drainage. This is the first published report of this connection, although varicella is known to be associated with abscesses in general. Practitioners should be aware that cervical abscesses may complicate varicella infections.

## 1. Introduction

Cervical abscesses are frequently diagnosed and treated by otolaryngologists. As head and neck surgeons, we may be tempted to perform a directed physical exam targeted to the site of symptoms. However, this case illustrates the need for a complete assessment of the patient and the importance of keeping an open mind with regard to parsimonious explanations of a constellation of signs and symptoms.

## 2. Case Presentation

A previously healthy 19-month-old Amish female presented to her primary care physician with a 5-day history of right neck swelling, decreased oral intake, and decreased urine output. She developed a rash on the day of presentation, which prompted her parents to have her evaluated. There was no history of recent travel or sick contacts. She had no previous immunizations and was otherwise healthy. A CT of her neck, ordered by the primary care physician, suggested a retropharyngeal abscess, and she was subsequently transferred to our tertiary care facility.

On examination, she was afebrile and breathing effortlessly. Torticollis was noted. There was firm induration of the right neck, measuring approximately 7 × 5 cm in diameter. She was found to have edema of the right side of her soft palate and peritonsillar region, as well as an erythematous fullness on the posterior oropharyngeal wall. She had 2+ tonsils, clear rhinorrhea with erythematous nasal mucosa, and normal tympanic membranes. A red macular rash was noted on her forehead, neck, abdomen, and back with accompanying vesicles on some of the lesions. Her lungs were clear to auscultation bilaterally. She had a grade I of VI flow murmur that disappeared when supine. No abdominal masses were palpable, and her bowel sounds were normal.

**Figure 1:**
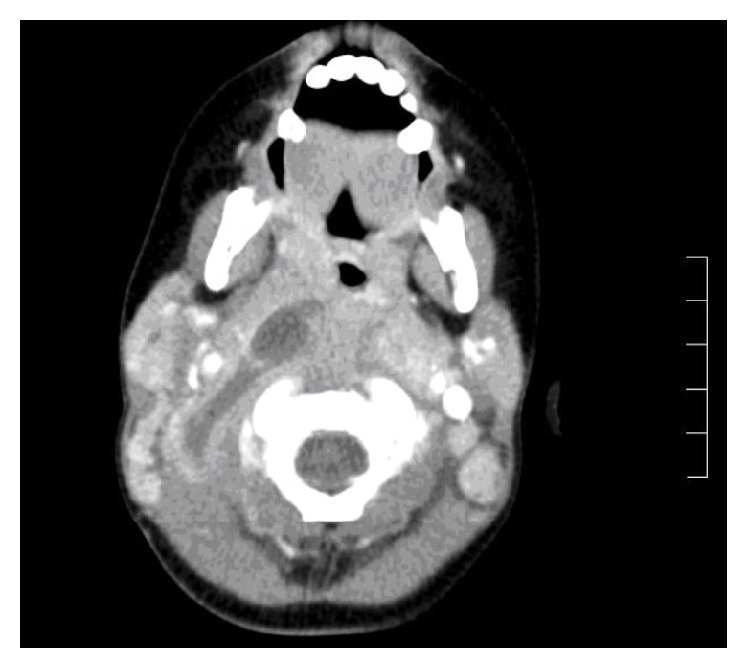
CT shows a hypodense area in the right parapharyngeal region, surrounded by an area of soft tissue thickening. There is minimal ring enhancement of the lesion.

The CT scan revealed a hypodense multiloculated mass consistent with an abscess in the right retropharyngeal space, extending from the soft palate inferiorly to the angle of the mandible (Figure 1).

She was admitted to the otolaryngology service and started on intravenous ampicillin-sulbactam. She was taken to the operating room the next morning for intraoral incision and drainage of the abscess. After induction of general anesthesia, a draining abscess was visualized on the posterior pharyngeal wall. An incision was made in the posterior pharyngeal wall under general anesthesia, but evacuation of pus was limited. An 18-gauge needle was used to aspirate several locations along the right side of her posterior pharyngeal wall from the nasopharynx to the hypopharynx. Bacteriological cultures from the abscess grew group A beta-hemolytic streptococcus. Intraoperative scrapings were taken of her skin lesions and returned positive for* Varicella zoster* antigen.

She was continued on IV antibiotics until postoperative day two, at which time she was switched to oral amoxicillin-clavulanate for a total of 10 days and discharged. Two weeks later, the family reported by telephone that her rash had resolved and that she had resumed a normal diet and activities.

## 3. Discussion

Complications of varicella infections differ greatly in their severity and in the tissues that they affect. Abscess formation is a known but relatively rare sequela of acute varicella infection [1, 2]. The location of abscesses in varicella is variable. A total of four patients with a history of varicella coincident with spinal epidural abscesses have been reported in the literature, with group A beta-hemolytic* Streptococcus* and* Staphylococcus aureus* as the underlying bacterial isolates from the abscesses in these cases [3–5]. Other cases of varicella complicated by staphylococcal and streptococcal abscesses on the extremities and back as well as in the mediastinum have also been reported [6–8]. To the best of our knowledge, no cases of primary abscesses in the cervical region in association with varicella infection in pediatric patients have been previously identified.

Varicella (chickenpox) is a highly contagious, typically benign disease that generally afflicts children. It is caused by the* Varicella zoster* virus, which is spread through direct contact with skin lesions or inhalation of viral particles. Varicella infection usually presents with pustular lesions covering the trunk and head. More than 90% of people are exposed to this virus by age twenty, with most cases observed in children less than ten years of age [9, 10]. Since the implementation of the varicella vaccine in 1995, there has been a significant reduction in the number of varicella cases and a 75% reduction in varicella associated hospitalizations [11]. One in five of those vaccinated will become infected, but the symptoms in these patients are much less severe [12, 13]. The patient described in this case had not received routine childhood immunizations, as this conflicted with the family's Amish belief system; however, this case underscores the importance of adhering to a correct vaccination schedule.

As with most infections, varicella can be complicated by spread of infection to other tissues of the body. Neonates, adults, and the immunocompromised are most vulnerable to complications of varicella [14]. The primary infection with the* Varicella zoster* virus creates a temporary depression of the immune system. This immune depression predisposes the individual to a secondary bacterial or viral infection [15, 16]. Some common complications of varicella include central nervous system dysfunction, pneumonia, and skin and soft tissue infections [10, 14, 17].

Along with these common complications of varicella, the immune depression caused by the primary varicella infection can also create a predisposition to tonsillitis [18]. Tonsillitis is an inflammation of the tonsils, which are lymphatic tissues located in the pharynx. Tonsillitis can be either bacterial or viral in origin. Group A beta-hemolytic* Streptococcus* is the most common bacterial cause [19, 20].

Tonsillitis is also associated with various complications, such as dehydration, airway obstruction, and abscess formation in the potential spaces of the neck. These potential spaces, created by fascial layers, are the retropharyngeal, vascular, submandibular, submental, parapharyngeal, peritonsillar, and parotid spaces [21]. Abscess formation in these potential spaces is commonly caused by tonsillar infection [22, 23].

The varicella infection seen in our patient may not have been the direct cause of her retropharyngeal abscess but still played a pivotal role in its formation. A combination of factors including the weakened immune system created by the primary varicella infection, the development of tonsillitis, and the proximity of the tonsils to the retropharyngeal space may have been vital in the development of the patient's abscess.

## 4. Conclusion

This case is the first report of a retropharyngeal abscess occurring as a sequela of varicella infection in a pediatric patient. It underscores the need for a complete physical examination as well as the need to consider parsimonious explanations when disparate signs and symptoms occur together.
